# Torsional stability of fixation methods in basicervical femoral neck fractures: a biomechanical study

**DOI:** 10.1186/s13018-024-04842-5

**Published:** 2024-06-22

**Authors:** Chantas Mahaisavariya, Surasak Jitprapaikulsarn, Banchong Mahaisavariya, Nattapon Chantarapanich

**Affiliations:** 1 Golden Jubilee Medical Center, Faculty of Medicine Siriraj Hospital, Mahidol University, Bangkok, Thailand; 2 Department of Orthopedic, Buddhachinraj Hospital, Phitsanulok, Thailand; 3 Department of Orthopedic Surgery, Faculty of Medicine Siriraj Hospital, Mahidol University, Bangkok, Thailand; 4 Department of Mechanical Engineering, Faculty of Engineering at Sriracha, Kasetsart University, Chonburi, Thailand

**Keywords:** Basicervical, Dynamic hip screw, Femoral neck fracture, Finite element analysis, Rotational instability

## Abstract

**Background:**

Basicervical femoral neck fracture is a rare proximal femur fracture with a high implant failure rate. Biomechanical comparisons between cephalomedullary nails (CMNs) and dynamic hip screws (DHSs) under torsion loading are lacking. This study compared the biomechanical performance of three fixations for basicervical femoral neck fractures under torsion load during early ambulation.

**Methods:**

The biomechanical study models used three fixations: a DHS, a DHS with an anti-rotation screw, and a short CMN. Finite element analysis was used to simulate hip rotation with muscle forces related to leg swing applied to the femur. The equivalent von Mises stress (EQV) on fixation, fragment displacement, and strain energy density at the proximal cancellous bone were monitored for fixation stability.

**Results:**

The EQV of the short CMN construct (304.63 MPa) was comparable to that of the titanium DHS construct (293.39 MPa) and greater than that of the titanium DHS with an anti-rotation screw construct (200.94 MPa). The proximal fragment displacement in the short CMN construct was approximately 0.13 mm, the greatest among the constructs. The risk of screw cutout for the lag screw in short CMNs was 3.1–5.8 times greater than that for DHSs and DHSs with anti-rotation screw constructs.

**Conclusions:**

Titanium DHS combined with an anti-rotation screw provided lower fragment displacement, stress, and strain energy density in the femoral head than the other fixations under torsion load. Basicervical femoral neck fracture treated with CMNs may increase the risk of lag screw cutout.

**Graphical abstract:**

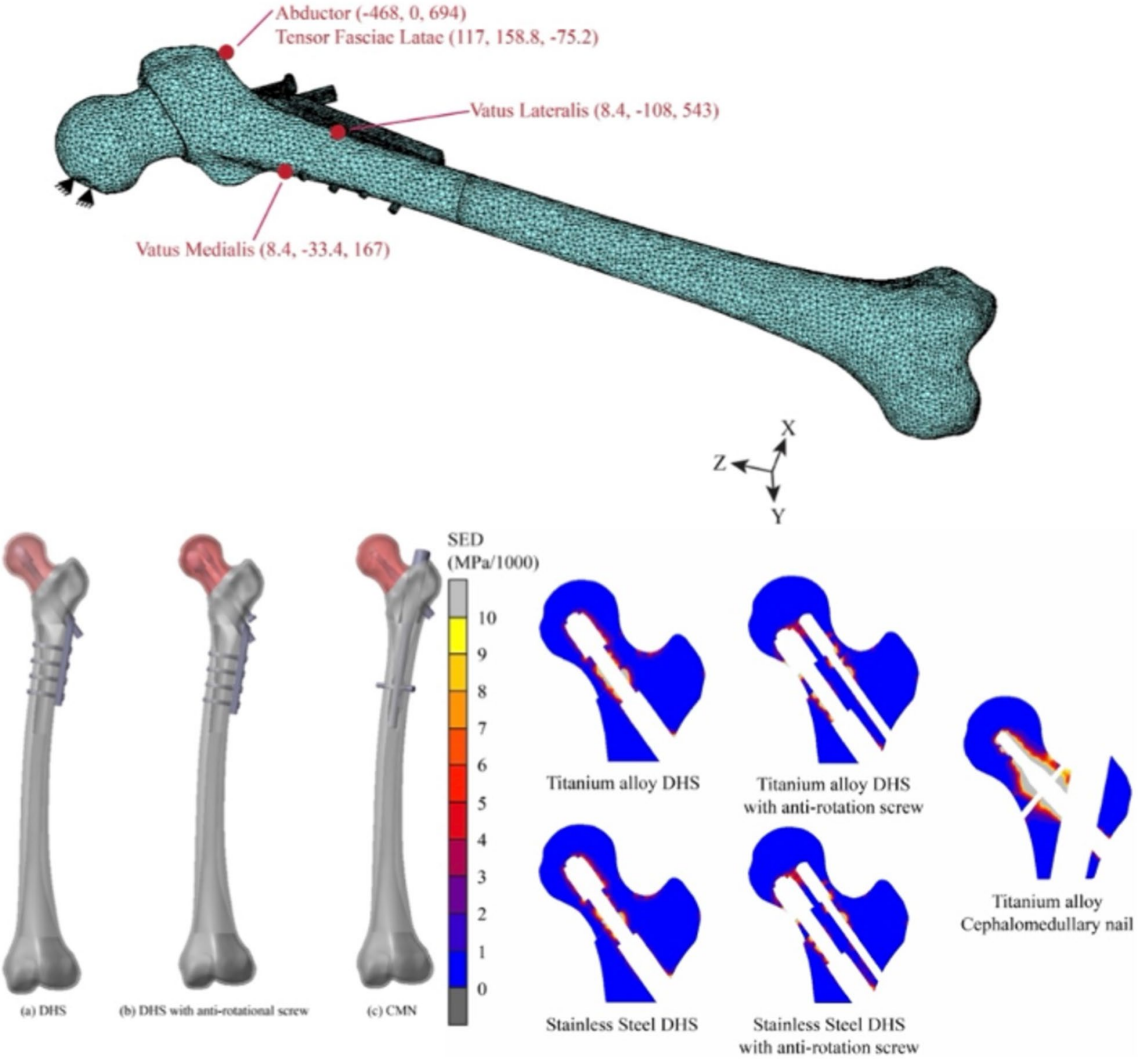

## Introduction

Basicervical femoral neck fractures, with a prevalence ranging from 1.8 to 7.6% [1], present challenges due to their rarity and potential instability, leading to high fixation failure rates compared to intertrochanteric fractures [1–3]. Despite the extracapsular location of the fracture site, surgical treatment remains challenging.

Cephalomedullary nails (CMNs), dynamic hip screws (DHSs), and multiple screws are the standard fixation devices for basicervical fractures. Complications, such as lag screw cutout, have raised concerns about CMN performance. Bojan et al.’s [2] large cohort retrospective study specifically reported screw cutout complications within 12 weeks after surgery. Similarly, studies by Mahaisavariya et al. [3], Watson et al. [1], and Lobo-Escolar et al. [4] observed screw cutouts, ranging from 6 weeks to 3 months after surgery, adding to the controversy regarding the efficacy of CMN in managing basicervical fractures.

During the gait cycle, screw cutout in CMNs is typically initiated by the vertical load from the body weight acting on the femoral head, leading to varus collapse of the proximal fragment. This collapse results in superior migration of the proximal screw fixation site and subsequent migrates through the superior part of the femoral head [5, 6]. Within the first six weeks after surgery, when full weight bearing is limited, the occurrence of screw cutout is reduced. However, screw cutout may still be caused by anterior–posterior torsion forces from hip rotation during the swing phase of the gait cycle, which is allowed immediately after surgery [7, 8]. The proximity of basicervical fractures to the capsular attachment, coupled with the absence of major ligamentous structures, reduces torque resistance and stability against torsion compared to intertrochanteric fractures. This is supported by the retrospective study of Bojan et al. [2], which suggested that screw cutout in CMNs predominantly results from torsional forces, leading to rotational displacement of the proximal fragment. These findings underscore the contribution of rotational forces around the hip joint to the incidence of screw cutout.

Previous research has focused primarily on biomechanical performance comparisons between DHSs and multiple screw fixation systems for femoral neck fractures [9–12]. In contrast, direct biomechanical comparisons between CMN and DHS fixations have not been performed. Existing biomechanical analyses have often emphasized walking and stair climbing under physiological loads, which included vertical weight bearing and associated muscle forces, when assessing bone-implant construct deformation and strength under these conditions [10–13]. However, the impact of torsion during early ambulation has not been adequately examined. This study introduces loading conditions that involve only anterior and posterior muscular forces, excluding body weight, aiming to address this research gap.

In this context, the study compared the biomechanical performance of DHS, DHS with an anti-rotation screw, and CMN in treating basicervical fractures during early ambulation. By evaluating the risk of screw cutout among these implants under torsional loading, this research offers critical insights for fixation selection and enhances awareness of potential complications in such scenarios.

## Materials and methods

This research received approval from the Institutional Review Boards of the authors’ affiliated institutions. Biomechanical analysis was conducted using the finite element (FE) method. For the geometric data, a femur model was obtained from one of the authors (NC), a trauma-free individual with no bony deformity who volunteered for scanning. The three fixations tested in this study were stainless steel and titanium DHSs, stainless steel and titanium DHSs with an anti-rotation screw, and titanium CMNs.

### Reconstruction of femur and fixation devices in three-dimensional models

Femur data acquisition was performed using a spiral computed tomography (CT) scanner (Philips Brilliance CT 64-slice, Philips, the Netherlands), with a slice thickness of 0.625 mm and a matrix size of 512 × 512 pixels. The data were stored in a Digital Imaging and Communications in Medicine (DICOM) file comprising a stack of two-dimensional transverse CT images. For three-dimensional (3D) reconstruction, these data were imported into 3Dslicer (slicer.org) [14], with Hounsfield units ranging from + 500 to + 2400 applied for thresholding the cortical bone layer of the femur, including the intramedullary canal. This thresholding was used to produce 3D polygon models of the femur, spanning from the proximal end to the mid-shaft, a length of 200 mm. The cancellous layer at the epiphysis was represented by a 3-mm offset from the cortical layer. These cortical and cancellous bone polygon models were then converted into 3D parametric models using computer-aided design (CAD) software (VISI, Hexagon AB, Sweden).

This study utilized three fixation methods: DHS (DepuySynthes, Oberdorf, Switzerland), DHS with an anti-rotation screw (DepuySynthes, Oberdorf, Switzerland), and short CMN (Zimmer Natural Nail, Cephalomedullary Asia, Zimmer Biomet, US). The DHS model comprised a 4-hole side plate (78 mm length, 135° barrel angle, 38 mm barrel length), a lag screw (110 mm length, 8 mm shaft diameter, 12.7 mm thread diameter), and a cortical screw. The anti-rotation screw was 6.5 mm in diameter and was cannulated and partially threaded. The short CMN featured a 15.5 mm proximal diameter, a 9.3 mm shaft diameter, a 180 mm length, a 130° CCD angle, a 15° anteversion angle, and a 4° proximal lateralization angle. The associated lag screw and distal screws were 10.5 mm and 5 mm in diameter, respectively. Dimensional measurements of the fixations were obtained using a digital microscope (Dino-Lite, AnMo Electronics Corp., Taiwan) and a Vernier caliper (Digimatic Caliper, Mitutoyo Co., Ltd., Japan). These measurements were subsequently used to reconstruct the 3D CAD models of the fixations with VISI software (Hexagon AB, Sweden).

**Fig. 1:**
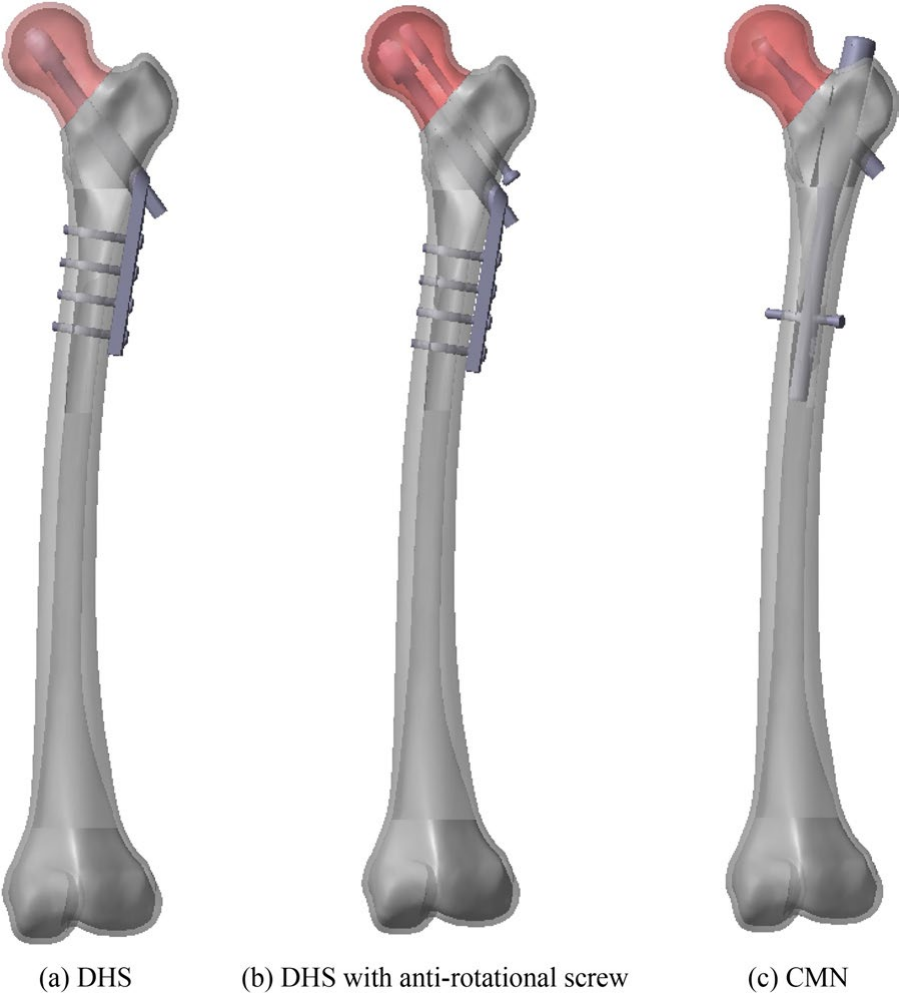
CAD model for bone-implant construct **a** DHS, **b** DHS with anti-rotational screw, and **c** CMN

A 50° Pauwels type II basilar neck fracture was simulated in the femur model. This fracture was oriented relative to the horizontal plane. Fixations were applied as per standard surgical techniques. For the DHS model, the lag screw was aligned parallel to the femoral neck axis, with its tip positioned 10 mm from the subchondral bone. The lag screw resided within the barrel, and the side plate was attached to the lateral cortical bone using four 4.5-mm cortical screws. In the DHS with an anti-rotation screw model, a similar technique was used, with the addition of an anti-rotation screw positioned just above the lag screw. Both techniques involved reducing the femoral head fragment into the adjacent shaft fragment. For the CMN model, the implant was inserted into the medullary canal of the femoral shaft, aligning the lag screw tip centrally to the femoral head, while the distal screw secured the nail to the cortical bone (Fig. 1).

### FE model generation

CAD models of the fractured femur and fixations were converted into mesh models for FE analysis utilizing preprocessing software (Patran, MSC Software, USA) with an automatic mesh generation function. Throughout the FE analysis, 4-node tetrahedral elements were used. The number of elements and corresponding nodes in each construct was determined through convergence analysis, focusing on monitoring the equivalent von Mises stress (EQV) on the fixation. Specifically, the DHS construct included 271,572 elements (66,994 nodes), the DHS construct with an anti-rotation screw had 281,619 elements (69,883 nodes), and the short CMN construct had 282,857 elements (69,921 nodes).

### Material properties

The material models were assumed to be homogeneous, isotropic, and linearly elastic, with all the material properties derived from the literature [15, 16]. For the cortical and cancellous bones, the elastic modulus were assigned as 17,000 MPa and 600 MPa, respectively, both with a Poisson’s ratio of 0.3. The stainless-steel DHS was allocated an elastic modulus of 200,000 MPa and a Poisson’s ratio of 0.30. Similarly, the titanium DHS and CMN exhibited an elastic modulus of 110,000 MPa and a Poisson’s ratio of 0.33.

### Boundary and contact conditions

**Fig. 2:**
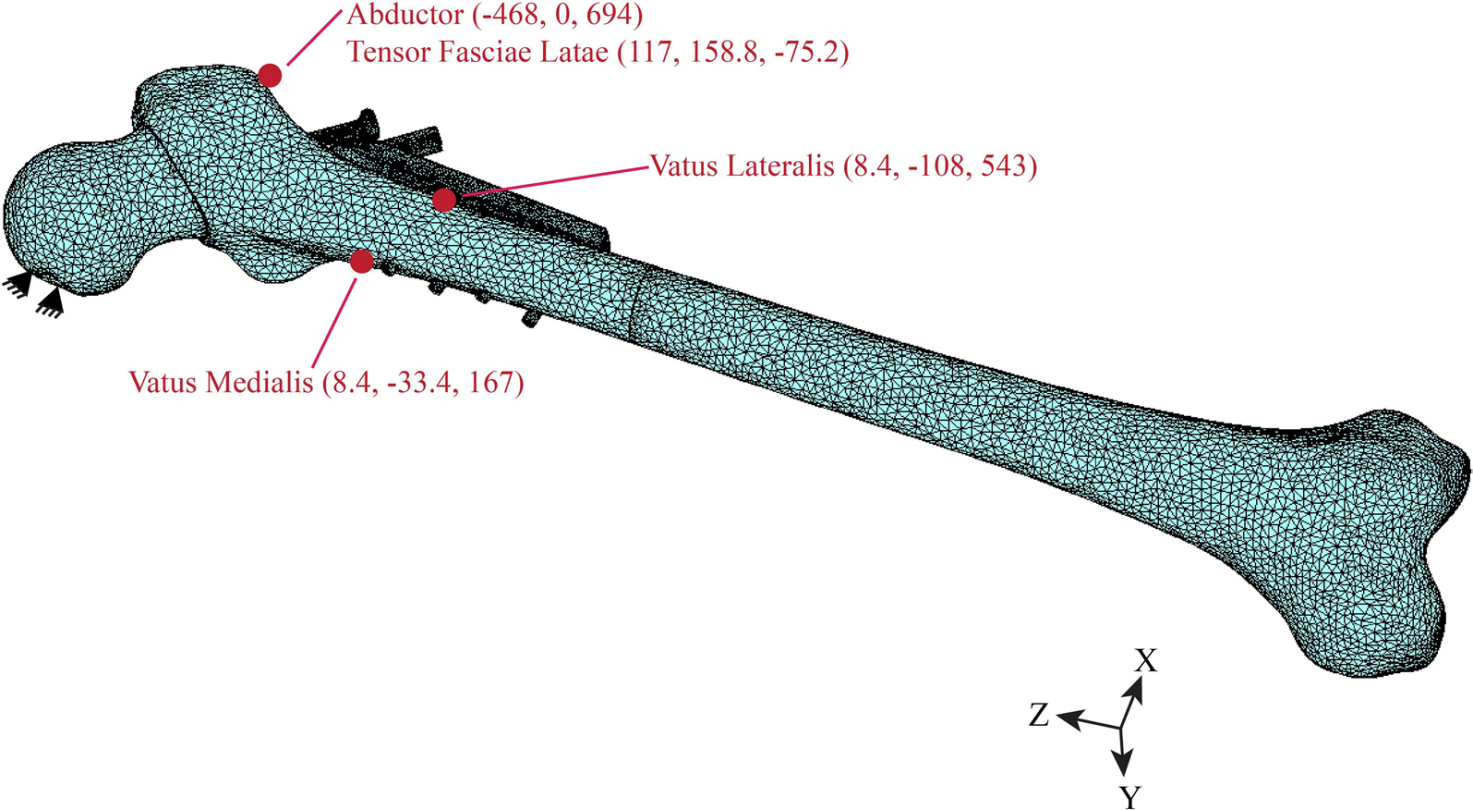
FE model with boundary condition

To simulate hip rotation, the FE model applied muscle forces related to leg swing to the femur, with the proximal femur constrained at the foveal capitis site. This setup is depicted in Fig. 2. Contact between cortical and cancellous bone was established, except at the reduced fracture interface, where the bones were allowed to interact. Fixation surfaces embedded in the bone were bonded to define complete interface fixation. For the interaction between different surfaces, relative translations were set, accompanied by specific friction coefficients for each interface: bone-titanium, 0.36 [15]; titanium-titanium, 0.30 [15]; bone-stainless steel, 0.15 [15]; stainless steel-stainless steel, 0.23 [15]; and bone-bone, 0.46 [13].

## Results

### Effect of the EQV on fixation

**Fig. 3:**
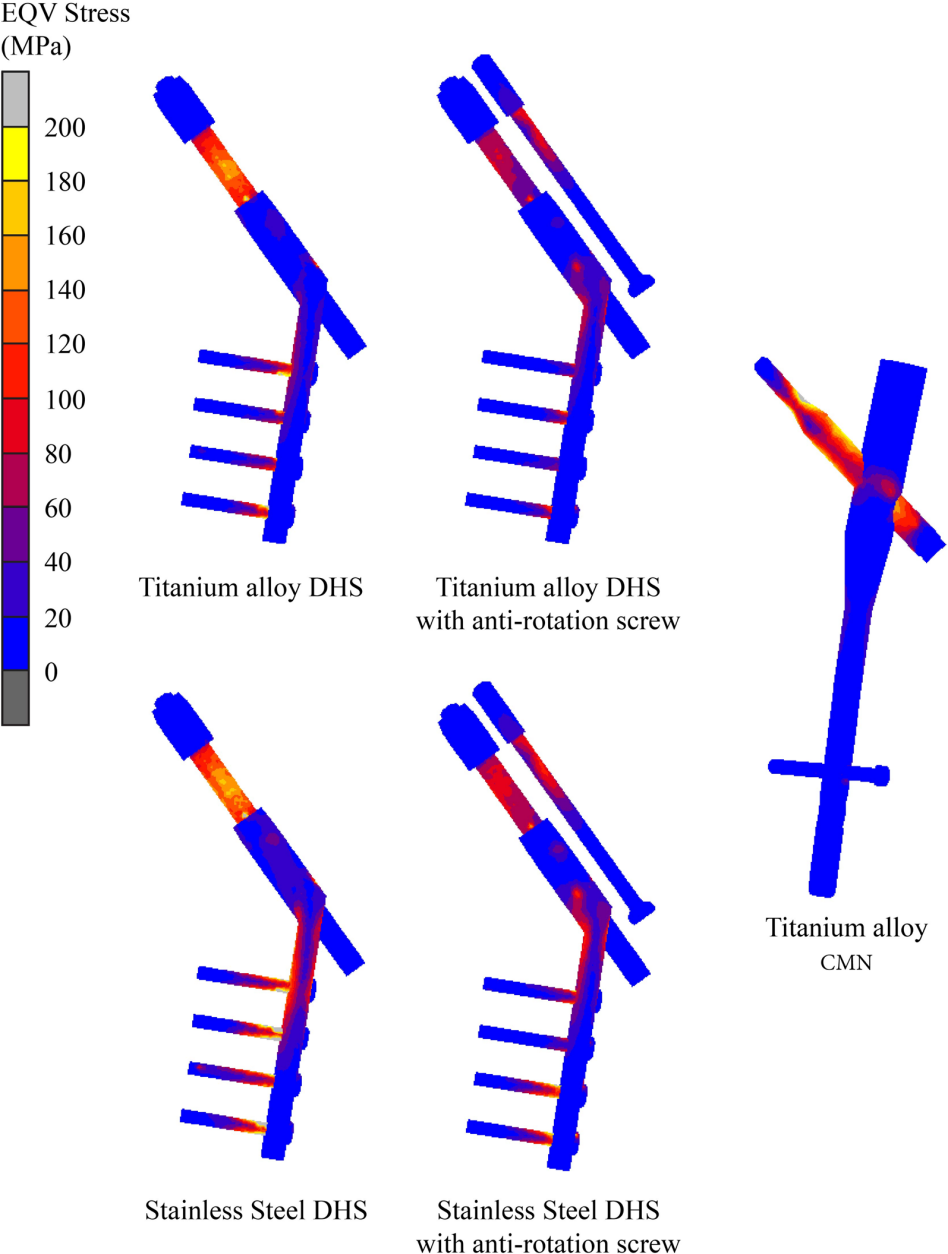
EQV stress on fixation

The EQV was localized on the lag screw/barrel interfaces for both the DHS and the DHS with anti-rotation screw constructs and at the lag screw/screw hole for the short CMN construct (Fig. 3). Stainless-steel DHS constructs exhibited higher EQVs (DHS: 378.88 MPa; DHS with an anti-rotation screw: 290.89 MPa) than did titanium DHSs (DHS: 293.38 MPa; DHS with an anti-rotation screw: 200.94 MPa). The incorporation of the anti-rotation screw in the DHS reduced the EQV by 23.2% to 31.5%. The short CMN construct demonstrated an EQV (304.63 MPa) comparable to that of the titanium DHS (293.39 MPa) but greater than that of the titanium DHS with an anti-rotation screw (200.94 MPa). The detailed data are shown in the Table 1.

**Table 1:** EQV stress on the implant, proximal fragment displacement, and strain energy density in the proximal cancellous bone following basicervical femoral neck fracture fixation

Fixation configuration	Fixation material	Implant stress (MPa)	Displacement of proximal fragment (mm)	SED at proximal cancellous bone (MPa/1000)
DHS	Titanium alloy	293.38	0.07	225.57
DHS with anti-rotation screw	Titanium alloy	200.94	0.06	215.14
DHS	Stainless steel	378.88	0.06	120.00
DHS with anti-rotation screw	Stainless steel	290.89	0.05	214.68
CMN	Titanium alloy	304.63	0.13	690.45

### Fragment displacement

Fragment displacement is a crucial indicator of fracture stability. The highest displacement in this study was observed for the short CMN construct. The proximal fragment was displaced by approximately 0.13 mm, marking the greatest displacement among all the tested constructs. Both the DHS and the DHS with anti-rotation screw models exhibited proximal fragment displacements ranging between 0.05 and 0.07 mm. Notably, no significant difference was observed between stainless steel and titanium DHSs or between DHSs and DHSs with anti-rotation screws.

### Strain energy density at proximal cancellous bone

**Fig. 4:**
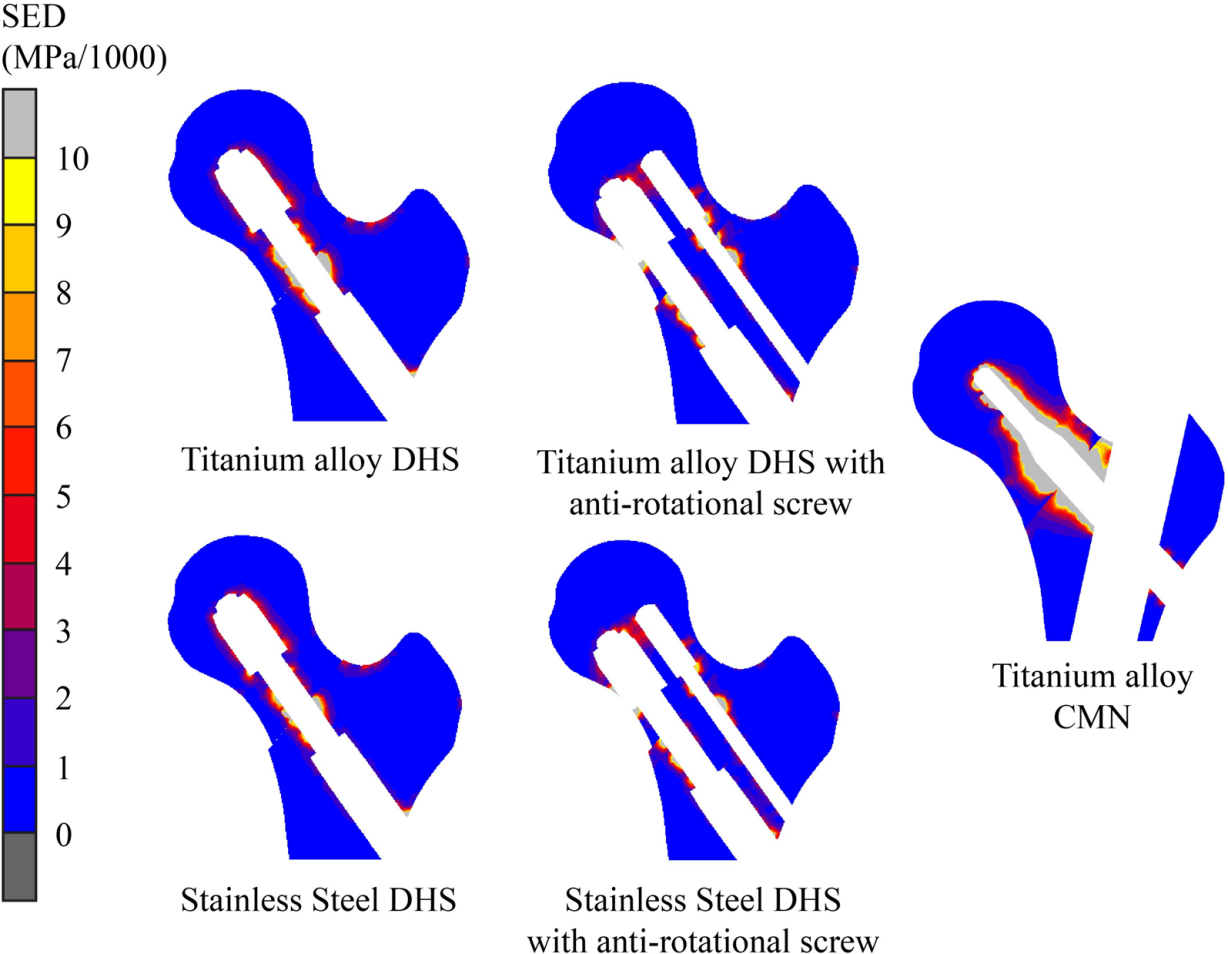
SED at proximal cancellous

Strain energy density (SED) assesses the attachment of a lag screw to cancellous bone. Higher SED values suggest an increased risk of screw loosening or cutout. Notably, the short CMN lag screw had a significantly greater risk of cutout than the other screw types, with an SED value of 690.45 MPa/1000. This value was 3.1 to 5.8 times greater than those of the DHS and of the DHS with anti-rotation screw constructs. In the context of titanium DHS, adding an anti-rotation screw resulted in lower SED values at the proximal cancellous bone, a trend that was reversed in the case of stainless-steel DHS (Table 1 and Fig. 4).

## Discussion

In the treatment of basicervical femoral neck fractures, the selection between DHS and CMN fixation implants remains a topic of contention [17–19]. Given that this fracture configuration does not involve the lateral wall of the proximal femur, DHSs and CMNs are considered appropriate options. Nevertheless, there is an ongoing need for more comprehensive biomechanical comparisons and testing between these implants.

The use of an anti-rotation screw in the DHS results in marginally lower displacement in patients with hip torsion than in those without it. Notably, the displacement in the CMN is approximately twice as high as that in the DHS. The DHS design, which provides greater interfragmentary compression under rotational torque, allows for more effective load stress transfer than does the CMN, thereby offering enhanced rotational stability for basicervical femoral neck fractures that typically have minimal or no comminution [20]. While the stress levels on the implants are similar for both CMN and DHS, adding an anti-rotation screw to DHS significantly lowers this stress. Notably, the stress levels observed in all fixation types were well below the yield stress of their respective materials [21, 22], thus reducing the likelihood of fixation breakage under torsional loads.

The SED plays a pivotal role and is closely linked to the risk of screw cutout and long-term bone-implant instability after cyclic loading [23, 24]. Higher SED values are known to contribute to the failure of implant attachment to bone [24]. The short CMN construct was observed to have SED values in the proximal cancellous bone that were 3.1 to 5.8 times greater than those of the other fixation constructs. This result suggested a notably increased risk of screw cutout in the proximal femur. These results are also consistent with the clinical postoperative observations of Mahaisavariya et al. [3], who reported an increased risk of screw cutout with CMN for stabilizing basicervical femoral neck fractures. Consequently, in line with the results of previous studies, the findings of our current biomechanical investigation reinforce the recommendation to avoid using CMNs for treating basicervical fractures. Conversely, DHS combined with an anti-rotation screw, which provides interfragmentary compression and limits rotational movement between fragments, shows promising outcomes. This combination results in lower SED in cancellous bone, along with reduced implant stress and fragment displacement, reinforcing the potential benefits of using DHSs with an anti-rotation screw for enhanced stability in such cases.

The findings of the present study demonstrated that under torsional load, the DHS outperformed the CMN, underscoring the enhanced torsional strength provided by the addition of an anti-rotation screw to the DHS. Although previous retrospective clinical studies may not have confirmed the advantage of adding an anti-rotation screw to DHSs [25, 26], the present investigation highlighted its significant impact on enhancing torsional strength. These findings align with a biomechanical study on surrogate proximal femurs under combined axial and torsional loads [5]. Specifically, the titanium DHS showed a lower EQV and fragment displacement but a slightly greater SED at the proximal cancellous bone than did the stainless-steel DHS, suggesting a preference for titanium.

The approach of the present study, in which hip rotation was simulated through torsional load, contrasts with previous biomechanical evaluations that primarily applied vertical force at the femoral head or physiological loads [10–13, 17]. Previous methods were unable to visualize the torsion effect created by hip rotation, a critical aspect for understanding implant performance. The current investigation focused on the effects of the bone-implant construct under torsion loading, which effectively mimicked hip rotation. This approach provides new insights into implant strength under conditions such as one-legged stances during walking and stair-climbing activities. It also offers a novel perspective not fully explored in prior studies, presenting a unique contribution to the field. To the authors’ knowledge, no other study has previously used this approach.

This study assessed the biomechanical efficacy of various fixation methods for basicervical femoral neck fractures in patients experiencing hip torsion. Between the 4-node and 10-node tetrahedral elements, the 4-node was selected for the study since previous studies have shown insignificant differences in results and also provide shorter computational time [26]. Despite yielding valuable findings, the study possesses inherent limitations. Primarily, the positioning of the lag screw was solely dictated by the surgical approach, disregarding existing literature that correlates lag screw placement in the femoral head with the risk of screw cutout [2, 27]. Furthermore, the FE analysis did not consider the thread shape of the lag screw, a critical factor that influences lag screw migration [6]. The analysis was also limited to CMNs equipped with a single lag screw, omitting the potential insights from configurations using double lag screws [13]. Additionally, the fracture simulation in this FE model is simplified by creating single-plane osteotomy on a single femur, which may not reflect the actual occurrence of bone fracture which may be multiplanar fracture configurations [28] and could fail to reflect anatomical variations across the population. Lastly, the model simplified bone composition to only cortical and cancellous types, neglecting the heterogeneity seen in pathological conditions such as osteoporosis, where bone properties are significantly altered. These limitations highlight areas for further investigation and underscore the need for comprehensive research that incorporates these factors for a more in-depth understanding of fixation strategies in treating basicervical femoral neck fractures.

## Conclusion

This study involved a biomechanical assessment of three fixation methods for basicervical femoral neck fractures: stainless-steel/titanium DHS, stainless-steel/titanium DHS with an anti-rotation screw, and titanium CMN. The findings suggest that titanium DHSs with an anti-rotation screw demonstrate reduced fragment displacement, stress, and SED in the femoral head compared with those of other fixations under torsion loading. Furthermore, the study indicated an elevated risk of lag screw cutout in basicervical femoral neck fractures treated with CMN. These results offer valuable insights for clinical decision-making in the management of such fractures.

## Data Availability

The datasets used and/or analyzed during the current study are available from the corresponding author on reasonable request.

## Abbreviations

CMN: Cephalomedullary nail
DHS: Dynamic hip screws
FE: Finite element
CT: Computed tomography
3D: Three-dimensional
CAD: Computer-aided design
EQV: Equivalent von Mises stress
SED: Strain energy density

## References

[1] Watson ST, Schaller TM, Tanner SL, Adams JD, Jeray KJ (2016). Outcomes of low-energy basicervical proximal femoral fractures treated with cephalomedullary fixation. J Bone Joint Surg Am..

[2] Bojan AJ, Beimel C, Taglang G, Collin D, Ekholm C, Jönsson A (2013). Critical factors in cut-out complication after Gamma Nail treatment of proximal femoral fractures. BMC Musculoskelet Disord..

[3] Mahaisavariya C, Pradhan N, Riansuwan K, Tharmviboonsri T, Rugpolmuang L, Mahaisavariya B, Tantigate D (2022). Risk factor of proximal lag screw cut-out after cephalomedullary nail fixation in trochanteric femoral fractures: a retrospective analytic study. Siriraj Med J..

[4] Lobo-Escolar A, Joven E, Iglesias D, Herrera A (2010). Predictive factors for cutting-out in femoral intramedullary nailing. Injury.

[5] Kouvidis GK, Sommers MB, Giannoudis PV, Katonis PG, Bottlang M (2009). Comparison of migration behavior between single and dual lag screw implants for intertrochanteric fracture fixation. J Orthop Surg Res..

[6] Sommers MB, Roth C, Hall H, Kam BC, Ehmke LW, Krieg JC (2004). A laboratory model to evaluate cutout resistance of implants for pertrochanteric fracture fixation. J Orthop Trauma.

[7] Bergmann G, Graichen F, Rohlmann A (1993). Hip joint loading during walking and running, measured in two patients. J Biomech..

[8] Brown RH, Burstein AH, Frankel VH (1982). Telemetering in vivo loads from nail plate implants. J Biomech..

[9] Giordano V, Freitas A, Pires RE, Battaglion LR, Lobo MO, Belangero WD (2022). Evaluation of a locking autocompression screw model in pauwels type-3 femoral neck fracture. Vitro Anal Bioeng (Basel).

[10] Li J, Zhao Z, Yin P, Zhang L, Tang P (2019). Comparison of three different internal fixation implants in treatment of femoral neck fracture-a finite element analysis. J Orthop Surg Res..

[11] Samsami S, Augat P, Rouhi G (2019). Stability of femoral neck fracture fixation: a finite element analysis. Proc Inst Mech Eng H.

[12] Zeng W, Liu Y, Hou X (2020). Biomechanical evaluation of internal fixation implants for femoral neck fractures: a comparative finite element analysis. Comput Methods Programs Biomed..

[13] Komatsu M, Iwami T, Kijima H, Kawano T, Miyakoshi N (2022). What is the most fixable intramedullary implant for basicervical fracture and transcervical shear fracture?-A finite element study. J Clin Orthop Trauma..

[14] Fedorov A, Beichel R, Kalpathy-Cramer J, Finet J, Fillion-Robin JC, Pujol S, Bauer C, Jennings D, Fennessy F, Sonka M, Buatti J, Aylward S, Miller JV, Pieper S, Kikinis R (2012). 3D Slicer as an image computing platform for the Quantitative Imaging Network. Magn Reson Imaging..

[15] Chantarapanich N, Sitthiseripratip K, Mahaisavariya B, Siribodhi P (2016). Biomechanical performance of retrograde nail for supracondylar fractures stabilization. Med Biol Eng Comput..

[16] Mahaisavariya B, Chantarapanich N, Riansuwan K, Sitthiseripratip K (2014). Prevention of excessive medialisation of trochanteric fracture by a buttress screw: a novel method and finite element analysis. J Med Assoc Thai..

[17] Johnson J, Deren M, Chambers A, Cassidy D, Koruprolu S, Born C (2019). Biomechanical analysis of fixation devices for basicervical femoral neck fractures. J Am Acad Orthop Surg..

[18] Yoo JI, Cha Y, Kwak J, Kim HY, Choy WS (2020). Review on basicervical femoral neck fracture: definition, treatments, and failures. Hip Pelvis..

[19] Yoon YC, Kim CH, Kim YC, Song HK (2022). Cephalomedullary nailing versus dynamic hip screw fixation in basicervical femoral neck fracture: a systematic review and meta-analysis. Yonsei Med J..

[20] Paulsson J, Stig JC, Olsson O (2017). Comparison and analysis of reoperations in two different treatment protocols for trochanteric hip fractures - postoperative technical complications with dynamic hip screw, intramedullary nail and Medoff sliding plate. BMC Musculoskelet Disord..

[21] Maghami MH, Sodagar A, Zabihian A, Asgarian F. Implantable Biomedical Devices. 2012

[22] Sitthiseripratip K, Van Oosterwyck H, Vander Sloten J, Mahaisavariya B, Bohez EL, Suwanprateeb J (2003). Finite element study of trochanteric gamma nail for trochanteric fracture. Med Eng Phys..

[23] Chantarapanich N, Riansuwan K (2022). Biomechanical performance of short and long cephalomedullary nail constructs for stabilizing different levels of subtrochanteric fracture. Injury.

[24] Mahaisavariya B, Sitthiseripratip K, Suwanprateeb J (2006). Finite element study of the proximal femur with retained trochanteric gamma nail and after removal of nail. Injury.

[25] Voeten S, Deunk J, Vermeulen J, De Lange-De KE, van den Brand H, Zuidema W (2020). The addition of an anti-rotation screw to the dynamic hip screw. Acta Orthop Belg..

[26] Jiang D, Zhan S, Wang L, Shi LL, Ling M, Hu H, Jia W (2021). Biomechanical comparison of five cannulated screw fixation strategies for young vertical femoral neck fractures. J Orthop Res..

[27] Goffin JM, Pankaj P, Simpson AH (2013). The importance of lag screw position for the stabilization of trochanteric fractures with a sliding hip screw: a subject-specific finite element study. J Orthop Res.

[28] Zhan S, Jiang D, Hu Q, Wang M, Feng C, Jia W (2024). Single-plane osteotomy model is inaccurate for evaluating the optimal strategy in treating vertical femoral neck fractures: a finite element analysis. Comput Methods Programs Biomed..

